# Supplementary material for: Diabetic retinopathy as a predictor for peripheral compression neuropathies, a registry-based study
Source: PLoS One. 2022 Oct 13;17(10):e0275598. doi: 10.1371/journal.pone.0275598 (PMC9560219; doi:10.1371/journal.pone.0275598)
Supplement: Attachment — Submitted filename: Response to reviewers.docx [file pone.0275598.s001.docx]

Dear Editor and Reviewers,

Thank you for your comments. We address them below. Please see changes in the revised manuscript. We have also reviewed the formatting requirements and the reference list. We hope that you will find the revised manuscript suitable for publication.

On behalf of all authors,

Malin Zimmerman, MD, PhD

Reviewer #1: The purpose of the study was to investigate if retinopathy can be used as a factor predicting the development of CTS and UNE. The Authors have used a large cohort based on which the results obtained are convicting. The advantage of having access to 95,437 individuals from the National Diabetes Registry is promising to conduct some additional analysis based on correlation analysis of variables that authors have. It will be worthwhile to do such analysis on the available dataset to make the study more interesting for the groups working on diabetes and its complication. Therefore, it is suggested to perform correlation analysis among the variables to identify the factors that might be contributing to diabetes induced CTS and UNE.

Reply: Thank you for the suggestion. We have added a correlation analysis, please see table 4 and table 8.

Reviewer #2: Authors have presented the study well. Authors have focused on developing predictors of peripheral compression neuropathy using diabetic retinopathy. The study is adding knowledge to the currently available domain and is also useful for the countries like developing nations where the prevalence of diabetic patients is increasing.

**Reply**: Thank you! We hope that our study will help in the understanding of diabetes complications.
